# Autopsy in sudden unexplained death in youth: indispensable or in some cases redundant? An observational study

**DOI:** 10.1007/s00431-026-07286-7

**Published:** 2026-08-01

**Authors:** A. C. van der Gugten, T. M. Wemeijer, B. A. Semmekrot, R. B. J. Smit, C. Oostdam, W. L. J. M. Duijst, S. de Gier, E. van de Putte, PESUDIC collaborative, A. Custers, E. Edelenbos, J. Fuijkschot, B. Levelink, P.J. Puiman, R.R van Rijn, J.M. Ruskamp, B.A. Semmekrot, K.T. Verbruggen, H. Vlaardingerbroek, M.E. Wiesman

**Affiliations:** 1 Department of Paediatrics, University Medical Center Utrecht Wilhelmina Children’s Hospital, Utrecht, the Netherlands; 2 Faculty of Law, Department of Criminal Law and Criminology, Maastricht University, Bouillonstraat 1-3, 6211 LH Maastricht, The Netherlands; 3 Forensic Medicine, GGD (Public Health Service) Ijsselland, Zwolle, The Netherlands; 4 Department of Paediatrics, Canisius Wilhelmina Ziekenhuis, Nijmegen, the Netherlands; 5 Forensic Medicine, GGD (Public Health Service) Utrecht, Zeist, The Netherlands; 6 Forensic Medicine, GGD (Public Health Service) Fryslan, Leeuwarden, The Netherlands; 7 Department of Pathology, Division of Laboratories, Pharmacy and Biomedical Genetics, University Medical Center Utrecht, Utrecht, the Netherlands

**Keywords:** Autopsy, Child death review, SUDY, Forensic Medicine

## Abstract

Sudden Unexplained Death in Youth (SUDY) requires thorough investigation to identify underlying causes and guide prevention strategies. In the Netherlands, cases are investigated using the standardized Postmortem Evaluation of Sudden Unexplained Death in Infants and Children (PESUDIC), in which autopsy is offered as a standard component. However, its invasive nature and associated time burden may limit parental acceptance. This study evaluated to what extent the cause of death can be established using a limited set of diagnostic tests compared to the standard procedure including autopsy. In this observational study, children > 2 years of age who died suddenly and unexpectedly and underwent PESUDIC, including imaging and autopsy, were included. Two expert panels, consisting of a forensic and a pediatric specialist, independently assessed early diagnostic tests available before autopsy. Cases were classified by level of diagnostic certainty and need for autopsy. Panel conclusions were compared with the reference standard: a multidisciplinary audit incorporating all available information, including autopsy findings. Sixty-six cases were included (median age 12 years (IQR 4–14.7), 63% male). Panels identified indicative information for a cause of death in 60 patients (91%). Nevertheless, autopsy was required in most cases (*n* = 59) to confirm the diagnosis. In 7 cases (10.6%), panels were sufficiently confident to establish the cause of death without autopsy with complete agreement with the reference standard. Causes included obstructive gastrointestinal pathology (*n* = 5) and diabetic ketoacidosis with dehydration (*n* = 2).

*Conclusions*: Only a small proportion of children > 2 years of age with sudden unexpected death have a cause of death that can be established with sufficient certainty at an early stage. In the vast majority of cases, the cause of death remains uncertain, supporting the recommendation to perform an autopsy.

**Table:** 

**What is Known:** • *Autopsy in sudden unexplained death in youth is worldwide recognized as the gold standard for postmortem examination. However, its invasive nature and associated time burden may limit parental acceptance.*
**What is New:** • *In 7 of 60 sudden and unexplained deceased minors (10%), sufficient certainty regarding the cause of death can be obtained by other minimal invasive diagnostic test, so without the need of an autopsy. Causes without the need of an autopsy included obstructive gastrointestinal pathology and diabetic ketoacidosis with dehydration.*

**Supplementary Information:**

The online version contains supplementary material available at 10.1007/s00431-026-07286-7.

## Introduction

Sudden Unexplained Death in Youth (SUDY) refers to the sudden and initially unexplained—yet presumed natural—death of an individual under the age of 18, excluding perinatal and unnatural deaths. The unexpected death of a child without a clear cause has a profound emotional and psychological impact on both the family and healthcare professionals. In such cases, a comprehensive postmortem investigation may offer clarity regarding the cause of death and help identify potentially preventable factors [1].

In the Netherlands, SUDY is estimated to affect approximately 50 children each year [2]. These cases are investigated using the Dutch Postmortem Evaluation of Sudden Death in Infants and Children (PESUDIC) protocol, an extensive, stepwise diagnostic approach. The PESUDIC procedure includes a thorough review of the medical history, external physical examination, biochemical and microbiological analyses, radiological imaging, and full autopsy, and concludes with a multidisciplinary panel discussion to reach consensus on the cause of death [3]. The purpose of the procedure is to establish the cause of death, in order to contribute to the parents’ grieving process of the sudden loss of their child and to help identify potentially preventable factors.

Cases showing indications of an unnatural cause of death are excluded from the PESUDIC procedure. Parental consent is required for each step of the procedure. Between 2016 and 2021, the PESUDIC procedure was able to determine the cause of death in 58% of investigated children, while an additional 13% had a plausible cause identified [3].

The PESUDIC procedure had been preceded by a 2-year pilot of this post mortem procedure, between 2012 and 2013, called the NODO procedure (in Dutch: Nader Onderzoek DoodsOorzaak) which was evaluated for cost-effectiveness by Price Waterhouse Cooper [4].

To establish the cause of death in deceased children with certainty, it is essential that all diagnostic procedures required for a comprehensive evaluation are performed. Conventional autopsy by a trained pediatric pathologist is considered the gold standard for identifying the cause of death [5–8]. In the NODO procedure, an autopsy was offered as more or less mandatory and this resulted in 90% autopsies. In the PESUDIC procedure, 60% of the parents consented to autopsy [3]. Possible reasons for declining autopsy were recently investigated in a qualitative study and have also been described in other studies and include the invasiveness of the procedure, the time required to complete it, no perceived benefit from autopsy, religion, and fear of mutilation [9–12].

In recent years, alternative diagnostic methods such as postmortem whole-body magnetic resonance imaging (MRI) and computed tomography (CT) have been increasingly utilized in the investigation of sudden child deaths. However, studies conducted on relatively small cohorts, report wide ranges of concordance rates (18–83%) between imaging findings and autopsy results [13–15].

Also, previous research within the PESUDIC procedure stated that other diagnostic modalities like microbiological, metabolic, or genetic testing can be decisive in ascertaining the cause of death, when utility is being retrospectively assessed [16]. However, results from such diagnostic test are not immediately available, whereas decisions regarding whether or not to perform an autopsy must be made before these results are known. To date, it has not been systematically evaluated in which cases the cause of death can be established with sufficient certainty based solely on initial diagnostic results and whether this level of certainty is sufficient to support the omission of an autopsy in specific circumstances.

The aim of the present study is therefore to assess the diagnostic value of autopsy and to determine in which cases the cause of death can be reliably identified without need for autopsy.

## Methods

### Study population

Data were used from children who participated in the NODO procedure (2012–2013) and the PESUDIC procedure (2016–2022). Children aged 0–18 years were eligible for these procedures when the forensic medical examiner classifies the death as both unexpected, without signs of trauma or external cause, without an immediately identifiable cause, such as a known life-limiting condition or obvious signs of an acute fatal illness. A death is considered unexpected and unexplained when it occurs in a child who was previously healthy, had a stable chronic medical condition, or was experiencing only a mild acute illness that would not reasonably be expected to result in death. Cases involving stillbirth, peripartum death, or infants who had not yet been discharged home following birth are excluded from eligibility [3, 4, 10 ]. Diagnostic investigations following sudden unexplained death in children are conducted on a voluntary basis and participation requires parental consent for at least one component of the investigation. Parents can choose which components of the diagnostic process they consent to participate in. Most diagnostic procedures take place shortly after death, and autopsy is performed within 1 to 2 days postmortem, depending on local availability. We have focused on an age category (2 to 18 years) beyond Sudden Unexplained Death in Infancy (SUDI) in order to make the data from this study more comparable with that from studies of adults. Because imaging and autopsy were important diagnostic and outcome tests of the reference standard, only children who underwent imaging and autopsy were included.

### Data collection

Comprehensive and standardized data regarding medical history, postmortem physical examination, and diagnostic outcomes from the PESUDIC procedure were prospectively collected and systematically recorded in the local electronic health records of all Dutch University Medical Centers. A dedicated Castor database (Ciwit BV. Castor Electronic Data Capture. 2016. Version 2024.2.4.1, Amsterdam) was developed to capture diagnostic procedures and outcomes using standardized data collection forms. To ensure confidentiality and data protection, all data were anonymized before being entered into the national Castor Electronic Data Capture database, and only anonymized data were used for analysis. All diagnostic procedures were conducted in accordance with the national PESUDIC protocol, encompassing medical history, physical examination, laboratory testing, microbiological analysis, DNA sampling, imaging, and autopsy guidelines. Cases were reviewed by local multidisciplinary expert panels in accordance with the national PESUDIC protocol. This standardized protocol and data collection framework ensured harmonized procedures and data acquisition across participating centers [17]. The data used and the results presented in this study do not contain any duplicated data with previous publicized research.

### Outcome measures

Conclusions on the cause of death were made in a multidisciplinary audit as part of the PESUDIC procedure; this was the reference standard in our study. In the audit, all the diagnostic results, including microbiological results, toxicology, radiology, autopsy and, when applicable, metabolic and genetic results, combined with medical history and forensic external examination, were taken into account. A cause of death was categorized as explained, plausible, or unexplained. An explained cause had an abnormal postmortem finding corroborating with the history or a full explanation of the death provided by an indisputable postmortem finding. A cause of death was considered as plausible in cases where a postmortem finding did not fit with the medical history or vice versa. The cause of death remained unexplained in cases with an absence of history and postmortem findings related to a lethal condition. For the descriptions in this study, the explained and plausible categories were grouped together as having a determined cause of death.

Two expert panels, each comprising a pediatrician and a forensic physician (BS, EP, CO, RS), all experts in this field, independently reviewed the diagnostic test results for each patient available prior to the commencement of the autopsy. The diagnostic information provided to the panels included the medical history, postmortem physical examination, biochemical analyses (blood, spinal fluid, vitreous humor, urine), imaging studies, as well as rapid microbiological and toxicological tests. The panels were tasked with determining whether a presumed cause of death could be identified based on these data. *A presumed cause of death* was defined when at least one directional indication was identified in the medical history, external examination, or ancillary investigations. Additionally, they evaluated whether an autopsy was necessary due to uncertainty regarding the cause of death or that there was sufficient certainty about the cause of death. *Sufficient certainty* exits when the panel members identified clear indications of the cause of death based on the medical history and/or physical examination findings, potentially supported by laboratory results or radiological investigations. In cases of disagreement of the two panels on the need for autopsy, a third panel was consulted (JR, TW), and discussions continued until consensus was achieved. Panel conclusions were compared with the findings of the multidisciplinary audit.

### Statistical methods

For data analysis SPSS 28 for Windows (IBM Corp. Released 2021. IBM SPSS Statistics for Windows, Version 28.0. Armonk, NY: IBM Corp) was used. The data were summarized with descriptive analyses. The continuous variable age was assessed for normality using visual inspection of their distributions. As the distribution was found to be non-normal, continuous variables were summarized as median and interquartile range (IQR). Inter-rater agreement was assessed using Cohen’s kappa statistic. No further statistical analyses were performed due to the low numbers and variety of cases.

### Ethics

Parents were asked to provide informed consent for the use of postmortem data for scientific research purposes. In addition, the Medical Research Ethics Committee of the University Medical Center Utrecht (reference number WAG/mb/18/007175) determined that the Medical Research Involving Human Subjects Act does not apply to deceased individuals and therefore exempted this study from formal review.

## Results

**Fig. 1:**
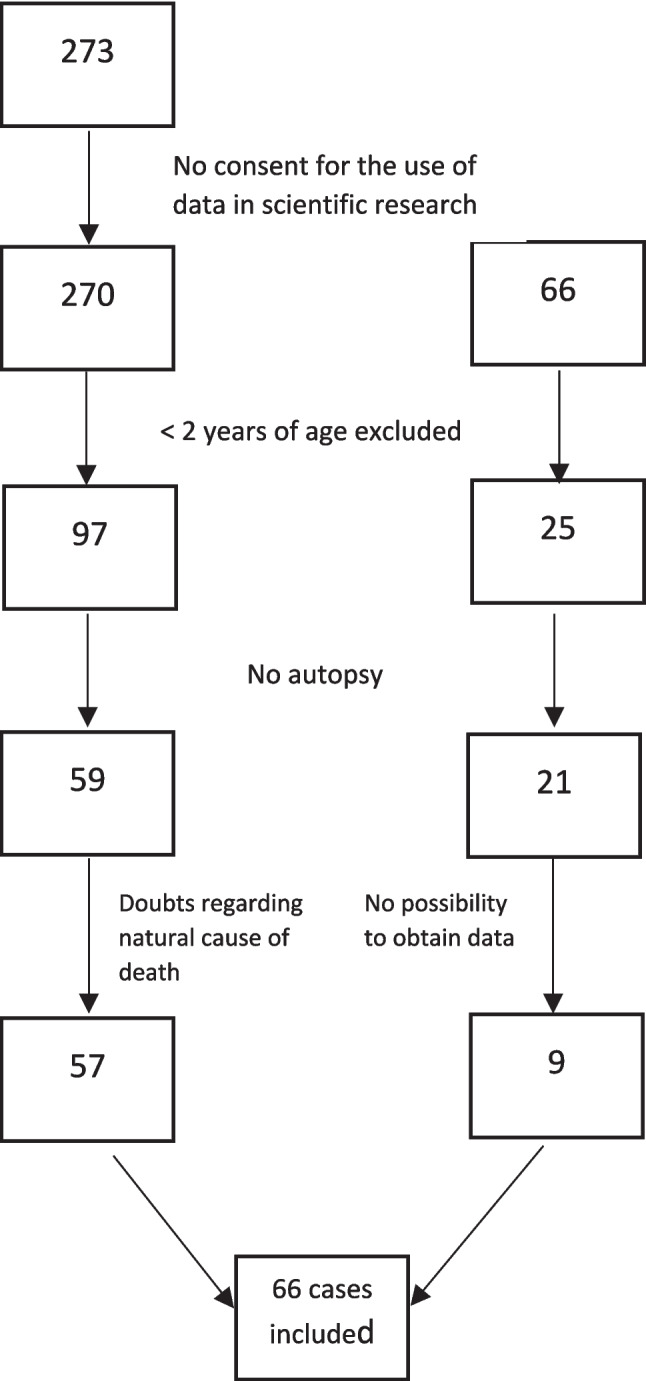
Flow diagram of case selection, including numbers assessed, excluded, reasons for exclusion, missing records, autopsy refusal, and final inclusion

Between 2012 and 2022, a total of 126 children above 2 years of age underwent the PESUDIC or NODO procedure and in 80 children the procedure included imaging and autopsy. From 12 patients who underwent the NODO procedure, it was impossible to obtain the data. In two cases, doubts arose regarding a natural cause of death during the procedure. These cases were excluded from this dataset. A total of 66 patients were included in the study (see Fig. 1 and Table 1 for more details).

**Table 1:** Basis characteristics of included cases and outcomes according to the reference standard, the multidisciplinary audit

Characteristics	Cases *n* = 66 (%)	
Sex—male	42 (63.6)	
Age—median (IQR) (years)	12 years (IQR 4–14.7)	
Age groups	2–4 years: 5–8 years: 9–12 years: 13–15 years: 16–18 years:	19 (28.7) 7 (10.6) 13 (19.7) 17 (25.8) 10 (15.2)
Cause of death certainty	Unexplained: Plausible: Explained:	11 (16.7) 6 (9.1) 49 (74.2)
Category cause of death determined*	Cardiovascular: Gastrointestinal: Neurological: Infectious: Other^$^:	20 (36.4) 10 (18.2) 3 (5.5) 16 (29.1) 6 (10.9)

*The explained and plausible categories were grouped together as having a determined cause of death

^$^Other were metabolic disorder, foreign body in main bronchus, renal insufficiency, diabetic ketoacidosis (three times)

The panel reviewed the results of all the diagnostic tests available in the 66 cases. A medical history, postmortem physical examination, and imaging (46 computed tomography, 27 MRI) were performed in all these children. Biochemical analysis was done in 95% of cases (*n* = 63, blood in 42, cerebrospinal fluid in 34, vitreous humor in 23, urine in 27). Toxicological rapid tests were performed in 44% of cases (*n* = 29) and microbiological rapid tests (nose and throat viral panel pcr) in 38% (*n* = 25).

The availability of diagnostic information varied between cases, reflecting routine clinical practice. A detailed overview of unavailable diagnostic information and the reasons for unavailability is provided in Supplementary Table 1.

The panels were able to identify a presumed cause of death in 60 patients (91%), while in 6 they were unable to determine any likely cause. Among the cases with a presumed cause, both panels agreed in 50 instances about the presumed cause (83%). Imaging, biochemical analysis, and history were the diagnostic tools most frequently providing valuable information for cause-of-death assessment. In 59 cases, panels expressed insufficient certainty regarding the cause of death and therefore deemed an autopsy necessary. In 7 cases (10,6%), both panels concurred that the cause of death was established with enough certainty to forgo an autopsy; of these, 2 initially required the involvement of a third panel to reach consensus. In all 7 cases, there was complete agreement between the panels’ determination and the definitive diagnosis established by the multidisciplinary audit following autopsy (see Table 2 for details). Overall agreement between the two panels regarding the need for autopsy was 97.0% (64/66 cases), corresponding to a Cohen’s *κ* of 0.83 (95% CI 0.66–1.00), indicating excellent agreement.

**Table 2:** Overview of causes of death according to the audit and the number of cases in which the panels considered an autopsy necessary or unnecessary

Category cause of death according to the audit	Panel, no autopsy necessary	Panel, autopsy necessary	Total
Cardiovascular	0	20	20
Gastrointestinal	5	5	10
Neurological	0	3	3
Infectious	0	16	16
Other	2	4	6
Unexplained	0	11	11
**Total**	**7**	**59**	**66**

The cause of death in these 7 cases were gastrointestinal obstructions in 5 cases (congenital mesenteric defect with sigmoid and small bowel herniation, intussusception, volvulus with lymphatic malformation as leading point, congenital mesenteric defect with small bowel intussusception and distal volvulus, strangulation with closed loop and internal herniation), and in the 2 other cases, a diabetic ketoacidosis debut with dehydration. In cases with a gastrointestinal cause of death, imaging proved to be the key diagnostic tool, whereas, in the two instances of diabetic ketoacidosis, biochemical analysis was crucial for diagnosis.

## Discussion

The aim of the present study was to evaluate the diagnostic utility of autopsy and to determine in which cases the cause of death can be established with sufficient confidence to forgo an autopsy. Our study demonstrates that at the time when a decision regarding autopsy performance must be made, there is sufficient certainty about the cause of death based on pre-autopsy diagnostic results in 10% of cases.

To the best of our knowledge, no similar study has been conducted before. In general, autopsy is considered to be the diagnostic test that offers the most certainty about the cause of death. In our national PESUDIC procedure, parents need to consent to every element of the procedure. Overall, approximately 60% of parents consented to an autopsy, of which only half agreed to include brain examination [3]. As mentioned previously, there appear to be several reasons for this. When parents do consent to autopsy, the practitioner will typically proceed with the examination, regardless of their professional opinion on its necessity. To make an informed decision and to properly counsel parents, it is essential to clearly establish in which cases an autopsy is not required to reach a definitive conclusion regarding the cause of death.

Based on the findings of this study, it can be concluded that when there is a documented history of new-onset diabetes mellitus confirmed by clinical chemistry results (in blood, cerebrospinal fluid of vitreous humor), performing an autopsy does not provide additional diagnostic value. Similarly, in cases where a clear obstructive gastrointestinal cause of death is evident from the medical history and imaging, an autopsy is unlikely to contribute further.

Our previous study [16] showed that autopsy provided a decisive cause of death in almost half of the cases it was performed. Although the autopsy plays an important role in excluding certain causes, this means that the other half of all causes determined were identified through other investigations than autopsy, like genetic testing or microbiological testing or combinations of these [3]. The discrepancy with our study is determined by the difference in methodology of our diagnostic study and by the fact that some results (e.g., genetic testing, blood cultures) are not available at the moment that a decision on whether or not to perform an autopsy has yet to be made. One consideration for the future is to allow more time before deciding to perform an autopsy, in some cases, for example by awaiting the results of microbiological diagnostics.

A limitation of our study is its observational design, including that not all investigations were performed in every case. During the expert panel assessments, all available diagnostic information was provided to the panel members. Missing diagnostic data could not be reliably imputed due to the nature of the study and the heterogeneity of the investigations performed. We did not observe evidence of a systematic pattern of missingness, and missing data appeared to be primarily related to the availability and performance of diagnostic tests in routine clinical practice.

Additionally, only patients who underwent both imaging and autopsy were included. Among the children who did not undergo autopsy (46 in total), a substantial proportion resulted from parental refusal. The reasons for declining autopsy were not systematically recorded but as we already described may include factors such as religious or cultural beliefs, the emotional burden on families, and time-related considerations. We do not expect these factors to be directly associated with the underlying cause of death and therefore do not anticipate a major impact on the overall study conclusions.

Some patients were excluded because no autopsy was performed, as the cause of death was considered already clear. Within the PESUDIC procedure, this rationale was documented for five children over the age of 2 years. Among these, four had an infectious cause of death, and one was attributed to midgut volvulus. The age distribution of the children who did not underwent autopsy was equal to the children included in the study. Regarding causes of death, an infectious cause was identified in nearly 50% of the children who did not undergo autopsy.

Assessments with panels are inherently subjective. To minimize this subjectivity, conclusions were only accepted when both panels independently reached the same judgment, or, in cases of disagreement, after review by a third panel. The accuracy of these assessments was subsequently evaluated against the audit findings, which served as the reference standard. Notably, in all cases for which the panels concluded that the cause of death could be established with sufficient certainty, this conclusion was consistent with the cause of death determined by the audit.

The external validity of our findings remains a consideration. In healthcare systems without access to structured postmortem diagnostic pathways, including systematic review of imaging and postmortem chemistry, the ability to establish a reliable cause of death based on clinical information alone is limited. In such settings, autopsy would therefore still be strongly indicated in most cases. Our findings may support the development of broader postmortem diagnostic strategies beyond autopsy alone, integrating clinical history with targeted ancillary investigations to improve cause-of-death determination.

## Conclusion

Our study demonstrates that autopsy remains crucial in the diagnostic evaluation of the cause of death in children who have died suddenly and unexpectedly without explanation. In a small subset of cases, autopsy can be omitted because the cause of death is already clearly established. This information might be helpful for physicians in asking for informed consent to autopsy in cases of child death.

## Supplementary Information

Below is the link to the electronic supplementary material.

ESM 1 (PDF 514 KB)

ESM 2 (DOCX 16.3 KB)

## Data Availability

All data supporting the findings of this study are available upon request. Other research that used these data is acceseble at: https://pubmed.ncbi.nlm.nih.gov/37852434/.
