# Supplementary figures and images for: Autopsy in sudden unexplained death in youth: indispensable or in some cases redundant? An observational study
Source: Eur J Pediatr. 2026 Aug 1;185(8):632. doi: 10.1007/s00431-026-07286-7 (PMC13428766; doi:10.1007/s00431-026-07286-7)

**Pesudic cases**

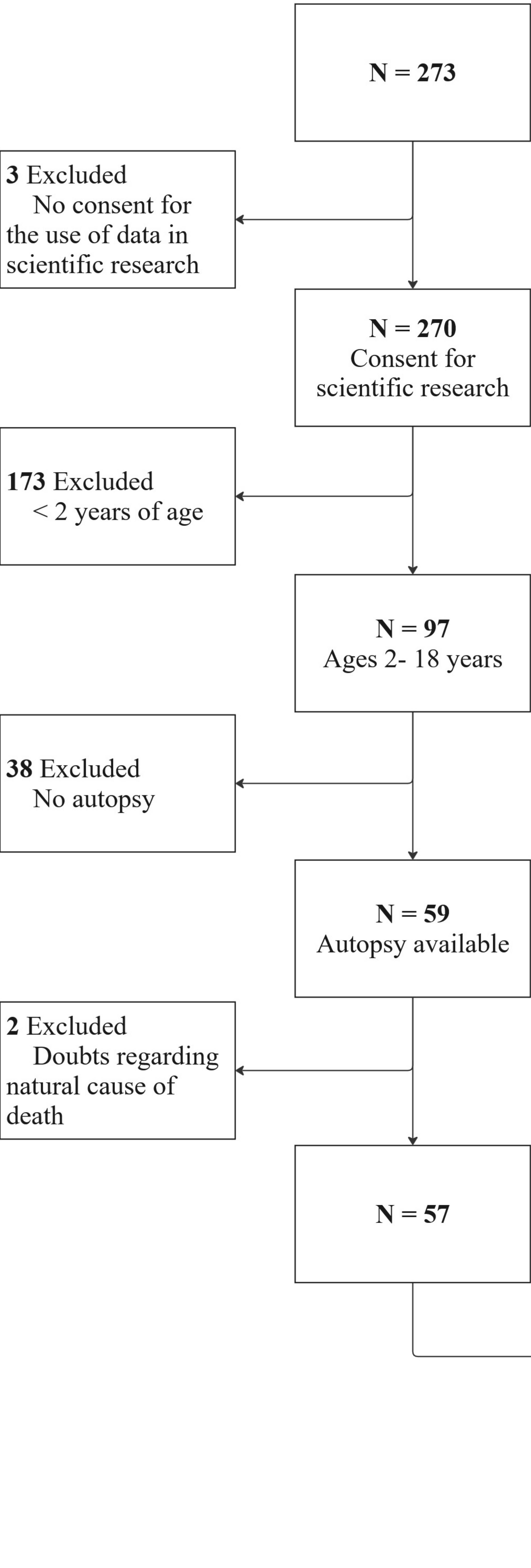

**NODO cases**

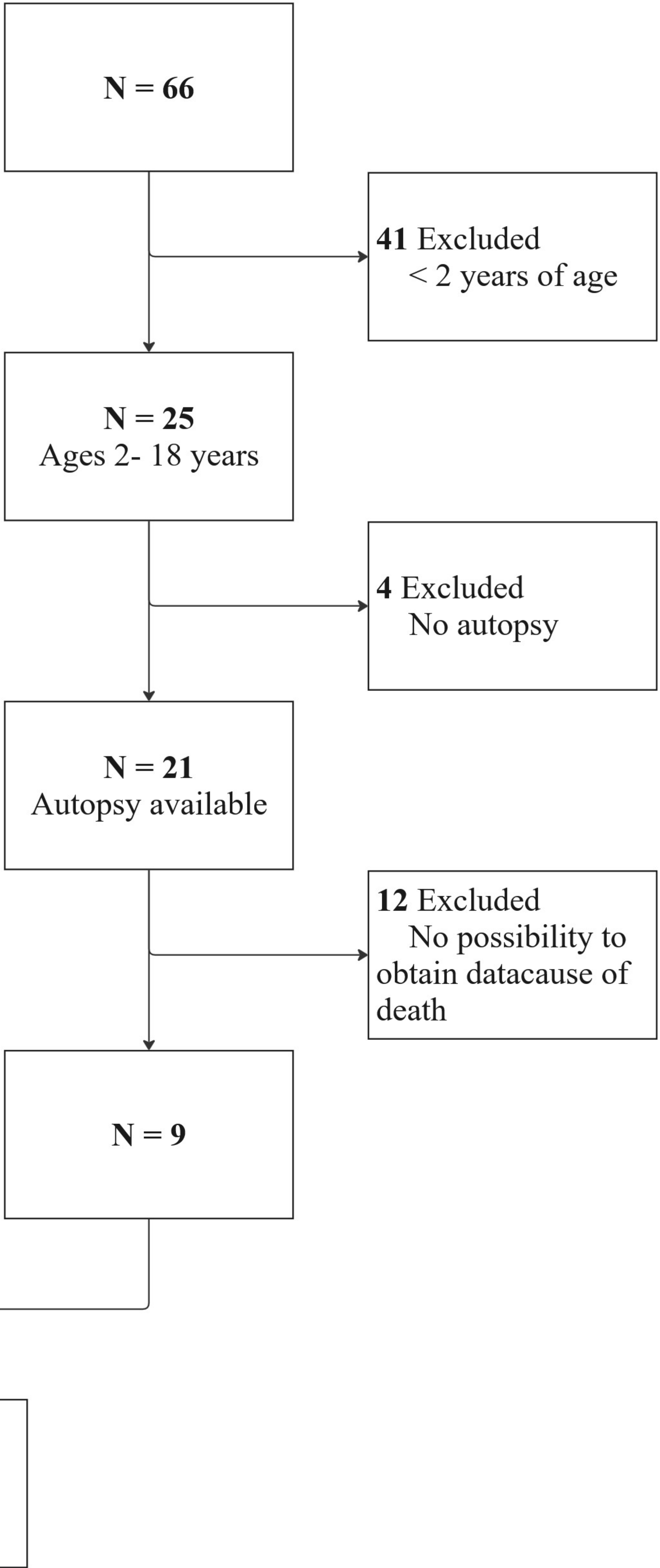

**N = 66**  
Final sample

Supplement: Supplementary file 1 — (PDF 514 KB) [file 431_2026_7286_MOESM1_ESM.pdf]
