# Supplementary material for: Autopsy in sudden unexplained death in youth: indispensable or in some cases redundant? An observational study
Source: Eur J Pediatr. 2026 Aug 1;185(8):632. doi: 10.1007/s00431-026-07286-7 (PMC13428766; doi:10.1007/s00431-026-07286-7)
Supplement: Supplementary file 2 — (DOCX 16.3 KB) [file 431_2026_7286_MOESM2_ESM.docx]

| **Variable/modality** | **Number (n)** | **Reason for unavailable information** |
| --- | --- | --- |
| No consent for use of data in scientific research | 3 in Pesudic | The reasons for declining consent were not recorded, and the mechanism underlying these missing data is unknown. |
| Age <2 years | 173 in Pesudic  41 in NODO | Predefined exclusion criterion. The study focused on children aged 2–18 years to distinguish this population from SUDI and improve comparability with studies in adults. |
| No autopsy performed | 38 in Pesudic  4 in NODO | The reasons for declining autopsy were not systematically recorded but may include factors such as religious or cultural beliefs, the emotional burden on families, and time-related considerations. We do not expect these factors to be directly associated with the underlying cause of death and therefore do not anticipate a major impact on the overall study conclusions.  In a few cases autopsy was declined because clinicians and parents already had a strong suspicion regarding the cause of death. These cases typically involved children with clinical and initial laboratory findings strongly suggestive of infection. While some degree of selection bias cannot be excluded, we expect the overall impact on the study conclusions to be limited. |
| Doubts regarding natural cause of death | 2 in Pesudic | The reason for exclusion was known; however, additional patient characteristics were not available for further assessment. |
| Excluded because no possibility to obtain data | 12 in NODO | These cases originated from a single hospital. The exclusion was related to inability to obtain the required data rather than patient-related factors; therefore, no association with patient characteristics was expected. |
| Not all diagnostic modalities were performed in every patient (medical history, postmortem physical examination and imaging were performed in all these children). | Availability varied by diagnostic modality  Biochemical analysis was done in 95% of cases  Toxicological rapid test in 44% of cases  Microbiological rapid test in 38% of cases | The decision to perform additional investigations was based on routine clinical practice and may have been influenced by clinical circumstances. Therefore, the availability of specific diagnostic modalities was not random. However, this reflects the real-world setting that was the primary aim of this study. |
